# SARS-CoV-2: An Update on Genomics, Risk Assessment, Potential Therapeutics and Vaccine Development

**DOI:** 10.3390/ijerph18041626

**Published:** 2021-02-08

**Authors:** Iqra Mehmood, Munazza Ijaz, Sajjad Ahmad, Temoor Ahmed, Amna Bari, Asma Abro, Khaled S. Allemailem, Ahmad Almatroudi, Muhammad Tahir ul Qamar

**Affiliations:** 1 Department of Bioinformatics and Biotechnology, Government College University, Faisalabad 38000, Pakistan; iqra6917@gmail.com (I.M.); munazzaijaz121@gmail.com (M.I.); 2 Department of Microbiology and Pharmacy, Abasyn University, Peshawar 25000, Pakistan; sahmad@bs.qau.edu.pk; 3 Institute of Biotechnology, Zhejiang University, Hangzhou 310058, China; temoorahmed@zju.edu.cn; 4 College of Informatics, Huazhong Agricultural University, Wuhan 430070, China; amnabari99@yahoo.com; 5 Department of Biotechnology, Faculty of Life Sciences and Informatics, Balochistan University of Information Technology Engineering and Management Sciences, Quetta 87100, Pakistan; asma.abro@buitms.edu.pk; 6 Department of Medical Laboratories, College of Applied Medical Sciences, Qassim University, Buraydah 51452, Saudi Arabia; K.allemailem@qu.edu.sa; 7 College of Life Science and Technology, Guangxi University, Nanning 530004, China

**Keywords:** SARS-CoV-2, pandemic, genomic characterization, pathophysiology, therapeutic strategies, COVID-19 vaccines

## Abstract

Severe acute respiratory syndrome coronavirus 2 (SARS-CoV-2) is a great threat to public health, being a causative pathogen of a deadly coronavirus disease (COVID-19). It has spread to more than 200 countries and infected millions of individuals globally. Although SARS-CoV-2 has structural/genomic similarities with the previously reported SARS-CoV and MERS-CoV, the specific mutations in its genome make it a novel virus. Available therapeutic strategies failed to control this virus. Despite strict standard operating procedures (SOPs), SARS-CoV-2 has spread globally and it is mutating gradually as well. Diligent efforts, special care, and awareness are needed to reduce transmission among susceptible masses particularly elder people, children, and health care workers. In this review, we highlighted the basic genome organization and structure of SARS-CoV-2. Its transmission dynamics, symptoms, and associated risk factors are discussed. This review also presents the latest mutations identified in its genome, the potential therapeutic options being used, and a brief explanation of vaccine development efforts against COVID-19. The effort will not only help readers to understand the deadly SARS-CoV-2 virus but also provide updated information to researchers for their research work.

## 1. Introduction

The emergence and re-emergence of pathogens is a global human health concern [1]. Coronaviruses are enveloped, their genomes are non-segmented, and they are single-stranded positive-sense RNA (+ssRNA) viruses belonging to the family Coronaviridae and order Nidovirales, which are widely dispersed in humans, animals, and birds. Coronaviruses cause various life-threatening diseases from respiratory infections to hepatic, enteric, and severe neurological diseases [2,3]. Six species of Coronaviruses were known to cause human diseases [4], out of which four (HKU1, NL63, 229E, and OC43) are widespread and responsible for the common cold in individuals with a weak immune response [4]. SARS-CoV-2 is the seventh coronavirus known to infect humans. Its exact origin is unknown; however, it shows homology with the previously identified coronavirus strains SARS-CoV (intermediate host, masked palm civet) and MERS-CoV (intermediate host, dromedary camel) [5,6]. The homology between SARS-CoV-2 and SARS-CoV is 82.45%, and the homology between SARS-CoV-2 and MERS-CoV is 69.58% [7]. SARS-CoV was responsible for SARS outbreaks in 2002–03 in Guangdong Province, China [8,9,10], while MERS-CoV was responsible for respiratory illness in the Middle East in 2012–13 [11]. The mortality rates of MERS and SARS were 37% and 10%, respectively [12,13]. SARS-CoV-2 triggered the COVID-19 pandemic, which spread rapidly worldwide and has become a public health concern [14]. In this review, we focus on SARS-CoV-2 novel genome organization, newly identified mutations in its genome, its transmission dynamics, clinical symptoms, potential treatment strategies, and recent advancements of vaccine production against COVID-19. 

## 2. Insights into Genomic Organization

Coronaviruses, which belong to the *Coronaviridae* family, are enveloped and pleomorphic viruses [15]. These are positive-sense RNA viruses with a genome size of 30 kb; which appears to be the largest size for a RNA virus, containing a 5′ cap and 3′ poly A-tail. Coronaviruses have a helical and flexible nucleocapsid. The membrane of these viruses contains a membrane glycoprotein, enveloped protein, and spike protein while the RNA is surrounded by nucleocapsid [16,17]. 

Virus RNA contains 6 open reading frames (ORF1ab, ORF3a, ORF6, ORF7ab, ORF8, and ORF10). Two-thirds of the virus genome comprises 1a/1b ORF and the remaining one-third of the genome code is used for M (membrane), S (spike), N (nucleocapsid), and E (enveloped) viral structural proteins [18,19].

**Figure 1:**
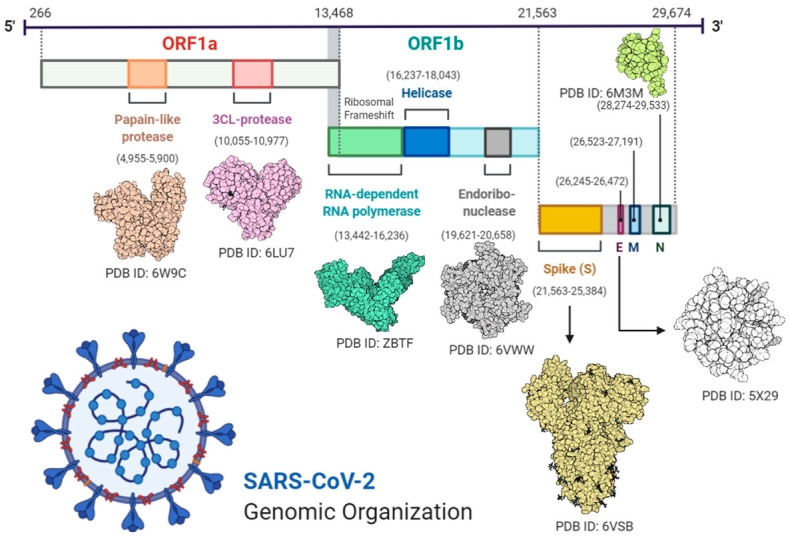
Complete structural and genomic organization of severe acute respiratory syndrome coronavirus 2 (SARS-CoV-2) [23].

Transcription was carried out by the synthesis of sgRNA (sub-genomic RNA) and replication-transcription complex (RTC), enveloped in double-membrane vesicles. Transcription termination occurred through transcription regulatory sequences that are present in between open reading frames (ORFs). There are 6 ORFs in the SARS-CoV-2 genome, as discussed above [18]. A frameshift mutation in ORF1a and ORF1b produces polypeptides (pp1a and pp1ab), which are further processed by virally encoded proteases such as main proteases (Mpro), chymotrypsin-like proteases (3CLpro), or by papain-like proteases for the production of non-structural proteins (nsps) [20,21]. Besides 1a and 1b open reading frames (ORFs), all other ORFs are responsible for the production of structural proteins (membrane, nucleocapsid, enveloped, and spike proteins), as shown in Figure 1. 

Through sequence analysis of SARS-CoV-2 and SARS-CoV, scientists proposed a mutation in the spike protein responsible for the jumping of the virus from animals to humans [22]. Similarly, some mutations have also been found in protein sequences which lead to the formation of proteins with a change in amino acid residues. For example, at position 723, instead of glycine there is a serine, while at position 1010 there is proline instead of isoleucine [22]. Potential disease recurrence depends on the evolution of the virus due to the accumulation of mutations in the viral genome over time.

### 2.1. Genome Sequencing

Through genomic sequence analysis, it has been confirmed that although SARS-CoV-2 has many similarities with SARS-CoV and other related coronaviruses, it is a novel virus (Table 1). The virus made a shift in the host organism from animals to humans with a few unique modifications/mutations. Genome sequence analysis suggests that most of the viral contigs/reads had a similarity with the genome of beta-coronavirus. SARS-CoV-2 has 96.20% and 88.00% levels of similarity to the previously published SARSr-CoV (RaTG13) and bat-SL-CoVZC45 genomes, respectively [3]. The sequencing of the SARS-CoV-2 genome from another study indicated 69.58% and 82.45% sequence similarity with MERS-CoV and SARS-CoV genomes, respectively [5,24]. Ten viral genome sequences obtained from 9 patients exhibited 99.98% sequence identity. In another study, sequences from eight patient samples had 99.98% sequence identity with each other across the whole genome [24]. BLASTn search of SARS-CoV-2 sequences has identified matches from the most closely related previously known viruses: SARS-like beta-coronavirus of bat origin, bat-SL-CoVZC45 (sequence identity 88%; query coverage 99%), and bat-SL-CoVZXC21 (sequence identity 88%; query coverage 98%). In 5 gene regions (7, M, N, 14, and E), sequence identity was more than 90% with 98.7% as the highest level for the envelope (E) gene. The Spike (S) gene demonstrated the lowest sequence identity of 75%. However, the sequence identity in 1a and 1b gene regions was 90% and 87%, respectively [24]. The majority of proteins encoded by SARS-CoV-2 were highly similar to proteins encoded by bat-related coronaviruses with a few insertions and deletions [24]. However, protein 13 and the S protein revealed 73.2% and 80% identity with other bat-derived viral proteins, respectively [25]. SARS-CoV-2 encoded a large spike protein, which is a major distinguishing feature among SARS-CoV-2, SARS-CoV, MERS-CoV, and other bat-derived coronaviruses. SARS-CoV-2 exhibits the same genomic organization as bat-SL-CoVZXC21, SARS-CoV, and bat-SL-CoVZC45, as revealed by comparison of predicted coding regions. Ten coding regions were identified including E, M, N, S, 10ab, 9, 8, 7, 3, and 1ab [24]. 

### 2.2. Phylogenetic Analysis

Phylogenetic analysis of SARS-CoV-2 genomes obtained from early patient samples suggested similarity in the sequence organization with beta-coronaviruses such as 5′ UTR (untranslated region), replicase complex (orf1ab), 4 genes (M, N, S, and E), 3′ UTRs (untranslated regions1), and some unidentified non-structural ORFs (open reading frames) [26]. Instead of having sequence similarity with beta-coronaviruses discovered in bats, SARS-CoV-2 is distinct from SARS-CoV, as well as MERS-CoV. Another piece of evidence pointing to its novelty is that the sequence identity in conserved replicase domains (ORF 1ab) is less than 90% between SARS-CoV-2 and other members of beta-coronaviruses and sarbeco-virus sub-genus of the Coronaviridae family [3].

### 2.3. Conserved Proteins

The S protein is responsible for membrane fusion and receptor binding. It is also critical in controlling virus transmission capacity and host tropism. The S protein of SARS-CoV-2 has two domains, namely the S1 and S2 domains. The S1 domain is responsible for receptor binding, while the S2 domain for membrane fusion [27]. It has been reported that a cellular protease (furin) is responsible for the cleavage of S1/S2 sites and this cleavage is necessary for the entry of virion in human lung cells and S protein facilitated cell fusion [28]. The S1 and S2 domains of SARS-CoV-2 have a sequence similarity of 93% and 68% with bat-SL-CoVZXC21 and bat-SL-CoVZC45, respectively [24,29]. Among sarbeco-coronaviruses, amino acid variations in S protein were identified. Although SARS-CoV and SARS-CoV-2 belong to different clades in the phylogenetic tree, they have 50 conserved amino acids in the S1 domain of the S protein. However, MERS-CoV has mutational differences in S proteins. Most of these mutational events occur in the C-terminal domain [24]. Several other proteases are also involved in different processes, such as entry of the virion, maturation of polyprotein, and assembly of different virion particles [30]. Other than the S protein, a variety of SARS-CoV-2 other proteins show similarity with proteins of other Coronaviridae family members, as shown in Table 2.

### 2.4. Receptor Binding Domain (RBD)

The RBD of SARS-CoV-2 is found in the C-terminal domain of spike protein as in SARS-CoV, Bat CoV HKU4, and MERS-CoV [32,33]. It was also reported that SARS-CoV-2 uses ACE2 (angiotensin-converting enzyme) as a cell receptor for entry into the human cells [34]. From the phylogenetic analysis, it was found that at genome level, SARS-CoV-2 is closely related to bat-SL-CoVZXC21 and bat-SL-CoVZC45, though the RBD of SARS-CoV-2 is highly similar to SARS-CoV. However, key residues of the receptor-binding domain responsible for the binding of the receptor were different in SARS-CoV-2 as compared to SARS-CoV [24]. From the above studies, it is again established that although SARS-CoV-2 has a great similarity with MERS-CoV, SARS-CoV, and some other bat-derived coronaviruses, it is a novel version of coronavirus and is responsible for an infection that is spreading globally.

## 3. SARS-CoV-2 Recent Mutations

RNA viruses have a high mutation rate but to survive, they achieve a balance of adaptions via mutations and replication-competent genome. For example, wild type SAR-COV reported showing less than one mutation in a sequenced genome (error frequency of 2 × 10^−5^), whereas SARS-CoV ExoN(−) revealed 10 mutations in a sequenced genome (error frequency of 3 × 10^−4^) [35,36]. Like other RNA viruses, SARS-Cov-2 has a high mutation rate and its multiple variants have been reported globally [37]. Research has revealed that 93 mutations were observed over the entire SARS-CoV-2 genome. In all major proteins, except for the envelope protein, 42 missense mutations were identified. There were 29 missense mutations in the polyprotein encoded by ORF1ab, 8 in the glycoprotein of the spike surface, 1 in the matrix protein, and four in the protein of nucleo-capsid. Spike surface glycoprotein has an important role in binding with host cells and regulates the host response [38]. It is also the key target of antibody neutralization [5]. Mutations in the glycoprotein present in the spike surface can induce some important conformational changes that can change antigenicity [39]. In another study, 5775 different genome mutations have been reported, including 2 in-frame insertions, 11 frameshift deletions, 36 stop-gained variants, 66 insertions in the non-coding region, 100 in-frame deletions, 142 deletions in non-coding regions, 484 mutations in non-coding regions, 1965 synonymous mutations, and 2969 missense mutations [40]. 

The UK faced a dramatic rise in COVID-19 cases that led to an increased number of epidemiological and virological surveys. The viral genome sequence has revealed that a significant number of cases belong to a newly reported phylogenetic cluster. The new variant of SARS-CoV-2 (B.1.1.7) has multiple spike protein mutations, (deletion 69–70, P681H, T716I, S982A, deletion 144, N501Y, D614G, A570D, D1118H) in addition to various mutations in different genomic regions [41]. Although it is clearly understood and predicted that viruses will continue to evolve due to mutations leading to the development of new variants, preliminary research in the UK indicates that this variant is substantially more infectious than its previous variants, having the potential of increasing the reproductive number by 0.4 or higher with increased transmissibility of up to 70 percent. This new variant appeared at the time of the year when social mixing has been increased traditionally. At this stage, there is no evidence of increased severity of infection linked with this new coronavirus variant [41]. This variant is also spreading in different countries throughout the world. Another variant known as B.1.351 has emerged independently in South Africa. It has many similarities with the B.1.1.7 variant and was detected in early October 2020. This variant has also spread to the USA and cases caused by this variant were reported there in early January 2021. A variant known as P.1 emerged in Brazil in early January 2021. It was first discovered in travelers during their regular screening in Japan. This variant has some additional mutations that may affect an antibody’s capability of identifying SARS-CoV-2. These newly identified strains seem to spread more rapidly. This is expected to eventually increase the coronavirus cases worldwide, which may result in a greater burden on hospitals and more deaths (Centers for Disease Control and Prevention).

## 4. Pathophysiology and Epidemiology

The recently identified SARS-CoV-2 is contagious and spreading globally with many confirmed cases. The infection caused by SARS-CoV-2 is transmitted by person-person contact via respiratory droplets [42]. The invasion of virion in different cells of the vascular system results in different inflammatory changes, such as necrotic changes, edema, and degeneration. It has been reported that SARS-CoV-2 infection also causes hypoxemia. It has been proven that the respiratory system is highly affected by this infection. Many neurologic symptoms have been associated with SARS-CoV-2 infection, such as agitation, dizziness, headache, confusion, and seizure [43].

Millions of people have been affected by SARS-CoV-2 and the mortality rate (number of deaths in a specific population with respect to population size during a specified time interval) is higher in males as compared to females, while individuals >65 years of age have also experienced a higher death ratio in comparison with younger adults and children. According to one study, the mortality rate is 62 times higher among individuals that are 55–65 years or higher, in comparison with individuals less than 54 years of age [44,45]. To date, there have been more than 200 countries affected by SARS-CoV-2 infection. The maximum number of cases has been reported in the USA (>25.3 million) followed by India (>10.7 million), Brazil (>9.9 million), and Russia (>3.7 million), while the death rate is higher in the USA and Brazil as compared to India and Russia (John Hopkins coronavirus Resource Center: https://coronavirus.jhu.edu/map.html (accessed on 1 February 2021). The mortality rate is highly variable among different countries, depending highly on health status, age statistics, and healthcare systems. In some countries, the mortality rate and active cases are increasing exponentially since the outbreak and are still at their peak. More than 100 million cases and more than 2.1 million fatalities have been reported so far across the world (John Hopkins coronavirus Resource Center).

## 5. Transmission Dynamics

Since the symptoms and features of SARS-CoV-2 were similar to pneumonia, this created confusion in the recognition of this novel coronavirus at the early stages. However, when disease conditions did not improve, physicians and doctors realized that something was wrong with diagnosis and treatment. Gradually, an increase in the patient count was reported, new cases were increasing day by day, and no treatment options were helping. The number of infected people kept on increasing and upon screening and testing, it was confirmed that this novel disease is pneumonia-like but is not pneumonia. There was a different viral agent behind the entire situation. A gradual increase in cases was due to late identification and reporting of diseases. 

Over time, it was identified that the virus spreads from human to human through close contact (from breathing, sneezing, and coughing). One study revealed that old people are more prone to infection caused by SARS-CoV-2 than young individuals and children. The age range of 425 early-infected individuals in Wuhan, China was 15–89 years. Out of 425 patients, 240 were males (almost 56%), and the rest were females [46]. The literature revealed the estimated mean incubation period of SARS-CoV-2 was 5.2 days. The mean duration from onset of disease to the first medical visit of patients was 12.5 days. The doubling period and epidemic growth rate was 7.4 days and 0.10/day, respectively [46]. Delayed disease recognition caused a deficiency of isolation systems for patients. Not practicing social distancing has paved the way for SARS-CoV-2 to spread all over the world and for the transmission of infection among millions of people. The death toll has risen, quickly triggering a pandemic.

## 6. Clinical Symptoms

Clinical symptoms of SARS-CoV-2 infection include coughing, fever, and shortness of breath/difficulty in breathing followed by severe outcomes like sepsis, ARDS (acute respiratory distress syndrome), acute kidney injury, and acute cardiac injury [47]. Clinical manifestations/symptoms of SARS-CoV-2 are divided into 3 groups according to the severity of the disease:

### 6.1. Mild Disease

Mild disease is characterized by mild pneumonia/non-pneumonia. This situation occurred in 80% of the SARS-CoV-2 cases [48].

### 6.2. Severe Disease 

Characteristics of severe disease are dyspnea, blood oxygen saturation (SpO2) ≤ 93%, a high PaO2/FiO2 ratio or P/F [partial pressure of oxygen, (PaO2), a fraction of inspired oxygen, FiO2)] < 300, and/or lung infiltrates >50% within 24 to 48 h; severe disease occurred in 10% of cases and was affected by patients’ respiratory frequency (>30/min) [49]. The mortality rate of SARS-CoV-2 has varied greatly at 0.7–1.3% and sharply changes from <0.002% for children under 9 years of age to 8% in patients aged over 80 years [50,51]. 

### 6.3. Critical Disease

The critical disease is characterized by multi-organ dysfunction/failure, sepsis/septic shock, and respiratory failure. Multi-organ dysfunction occurred in 1.4% of cases [38,39]. Intubation is provided to critical patients that have exerted positive effects on patients and approximately 3.2% of patients of SARS-CoV-2 require intubation or invasive ventilation at some stage of their disease [52,53]. 

## 7. Duration of SARS-CoV-2 Replication

The duration and virus replication rate are significant factors to determine the transmission risk and to make the decision pertinent to patient’s isolation [47]. Viral RNA detection is more feasible and a quick option that is adopted well to routine and emergency diagnosis compared to virus isolation, so many laboratories have carried out viral RNA quantification and qualification tests as a possible marker for the identification of any infectious virus [54]. Previously, the duration of SARS-CoV and MERS-CoV RNA detection had been documented. In the case of SARS-CoV, viral RNA was detected in the respiratory sample from 5 days to 28 days of disease onset [55]. Similarly, MERS-CoV viral RNA persisted from 3 days to 21 days in a lower respiratory sample [56,57]. In the case of SARS-CoV-2, viral RNA was present in surviving patients for 14–24 days. However, in non-survivors, viral RNA was sustained until death occurred. This information is very important in deciding a patient’s isolation and medication. One study described the viral replication duration among surviving and non-surviving patients [47]. It was found that the median duration for viral shedding or replication was 20 days among survivors from disease onset. However, virus replication was continuously detectable among non-surviving patients until death. The shortest duration of viral replication observed was 8 days among surviving patients [47]. Similarly, the longest duration of viral replication observed was 37 days in immunosuppressed patients [47]. In severe influenza virus infection, extended viral shedding was associated with fatal outcomes. Delayed antiviral treatments (antiviral drugs or antibodies treatments) were an independent risk factor associated with almost all forms of virus detection for a long time [47,58].

## 8. Risk Factors

There are always risk factors associated with the severity of a disease. There are multiple risk factors, which range from age factors to various diseases from which the patient is already suffering [59,60]. This is relevant for SARS-CoV-2. Previous studies related to SARS-CoV and MERS-CoV revealed risk factors associated with these diseases. For example, Sequential Organ Failure Assessment (SOFA) and blood level of d-dimers were major risk factors for the fatal illness of patients infected by SARS [32,33]. SOFA (Sequential Organ Failure Assessment) is a good marker for sepsis/septic shock diagnosis that tells us about the degree and state of multi-organ dysfunction [61,62]. Bacterial infections mainly cause sepsis, but some viral infections also lead to septic shock. Previous studies revealed that 40% of individuals with pneumonia had sepsis [63]. Similarly, age was also a major risk factor in patients affected by MERS and SARS [64,65]. A study was carried out to determine risk factors associated with SARS-CoV-2 [47]. The results suggested that people with a concomitant MERS or SARS status are more susceptible to SARS-CoV-2 than normal individuals. Concomitance was common in half of the patients, with the most common concomitant factor found to be hypertension followed by coronary heart diseases and diabetes [47]. Age is also a major factor since due to faulty function of T-cells and B-cells, excessive production of cytokines in older age leading to poor control on viral replication, and an extended pro-inflammatory response resulting in deadly outcomes [66]. The research included 191 patients, from which 56 died and the remaining patients recovered from the disease. The median age of the abovementioned 191 patients was 56; however, the overall age ranged from 18–87 years for the majority of the male population. It is also determined that > half of patients had sepsis [47]. Carcinoma, chronic kidney diseases, and chronic obstructive lung disease were also present in 2, 2, and 6 patients out of 191, respectively [47]. The risk factor data are summarized in Table 3.

Apart from age and comorbidity, lymphopenia, leukocytosis, elevated ALT, procalcitonin, lactate dehydrogenase, creatine kinase, high-sensitivity cardiac troponin I, prothrombin time, d-dimer, creatinine, serum ferritin, and IL-6 were also associated with mortality outcomes. Lymphocyte counts were low in patients with SARS-CoV-2. Surviving patients have a low number of lymphocytes with the onset of the disease but with hospitalization, the lymphocyte number improved. On the other hand, patients who died had severe lymphopenia with a very low number of lymphocytes [47]. Similarly, non-surviving patients had high levels of d-dimer, IL-6, high-sensitivity cardiac troponin I, lactate dehydrogenase, and serum ferritin throughout the clinical course, which increased with the worsening of illness as compared to for patients who survived. It was also seen that a high SOFA number, older age, and a blood d-dimer presence >1 μg/mL were associated with a large number of unexpected deaths [47]. Heart diseases such as new or worsening arrhythmia, myocardial infarction, and new or worsening heart failure were usually seen in patients with pneumonia. In 3% of patients with pneumonia, cardiac arrest occurred [67]. Apart from pneumonia, age, and pre-existing heart problems are usually associated with cardiac failure [68]. In viral respiratory infections and influenza, coronary heart diseases are associated with acute cardiac events [47,69]. In more than half of SARS-CoV-2 patients who died, elevated high-sensitivity cardiac troponin I was found. The reason was that increased d-dimer concentrations (>1 μg/mL) in the blood led to coagulation [47]. Previous studies also suggested that 90% of the patients with pneumonia had high d-dimer concentrations leading to coagulation [70]. High d-dimer levels associated with 28 days of mortality in patients with sepsis was also reported [71]. A summary of these laboratory biomarkers and their relevant levels in survivors and non-survivors is mentioned in Table 4.

## 9. Treatment 

There are some vaccines available that are authorized/approved and protect against coronavirus, but no specific drugs are available for the affected persons to date. However, different combinations of medicines and therapies are used to treat patients. Antiviral and anti-inflammatory treatments have been utilized. Most treatments are supportive and symptomatic. As symptoms of this disease are similar to those of pneumonia, early diagnosis and control of symptoms would lead to valuable results. For critical patients, invasive mechanical ventilation, non-invasive ventilation, continuous renal replacement therapy (CRRT), Oseltamivir therapy, and extracorporeal membrane oxygenation (ECMO) are applied to patients with hypoxemia [59]. Immunoglobulin G and convalescent plasma are also given to patients according to the situation [72]. Patients with severe shortness of breath can be shifted to intensive care units (ICU).

### 9.1. Antiviral Treatments

Treatments against SARS and MERS will give us guidance for treating coronavirus patients with previously utilized antiviral medicines [73]. To date, no specific drugs have been discovered for treating coronavirus. Previously, for SARS-CoV and MERS-CoV, various antiviral drugs were used to treat the diseases. Examples include ribavirin, methylprednisolone, neuraminidase inhibitors (peramivir, oseltamivir, zanamivir), acyclovir, and ganciclovir [59]. However, these drugs are not proving beneficial for the treatment of SARS-CoV-2. Fortunately, HIV (Human immunodeficiency virus) protease inhibitors and nucleoside analogues have been identified as a potential treatment and can be used to treat SARS-CoV-2 patients until the discovery of a specific drug is achieved [74]. Currently, patients with severe disease symptoms are treated with systemic glucocorticoids (which have anti-inflammatory, vasoconstrictive and antifibrotic effects [75]) and vasopressors (which reduces the ventilation duration and shortens the shock period [59]). In the beginning, oseltamivir (a medicine used to treat influenza) was also used to treat patients with early diagnosis to control symptoms [76] but, it has been discovered now that it has no impact against SARS-CoV-2 [77]. Antibacterial drugs such as moxifloxacin (which interacts with SARS-CoV-2 Main protease enzyme [78]) and azithromycin (which has arrhythmogenic potential, so it is not recommended by WHO but some local organizations have approved it with chloroquine [79]) are also being given to the patients [59]. An antiviral drug, GS-5734 (remdesivir), was also used successfully in the US. Remdesivir is a nucleoside analogue that has antiviral ability against many RNA viruses. As SARS-CoV-2 is an RNA virus, remdesivir is used as a drug to treat patients with severe symptoms. Remdesivir has the potential to block RNA dependent RNA polymerase activity [80]. It also interferes with nsp2 polymerase and the exon proofreading mechanism [81]. Remdesivir grabbed worldwide attention due to its effectiveness against the current outbreak of SARS-CoV-2 in in-vitro experiments [82]. Thus, it was thought that it can be used as a potential drug to treat viral infection caused by SARS-CoV-2, but lately, it has shown no impact in the treatment of SARS-CoV-2 in clinical trials (World Health Organization). Chloroquine is another drug that had been used previously as an anti-malarial as well as for autoimmune disorders [83]. Chloroquine is known to glycosylate viral cell receptors and increase endosomal PH, which disrupt the fusion of viruses with host cells. Chloroquine also has antiviral and immune-modulating activities, acting as an autophagy inhibitor suppressing the release and production of IL-6/ TNF-alpha. This drug is widely distributed in lungs and other parts of the body through an oral intake. One experiment carried out to check chloroquine activity demonstrated that chloroquine could play active role at entry and post-entry stages of SARS-CoV-2. Chloroquine is an economic and safe drug that has been in use for 70 years. It can be a potentially applicable drug to treat SARS-CoV-2 infections [84]. Other research revealed improved clinical outcomes in patients treated with chloroquine [85,86,87]. On the other hand, hydroxychloroquine has proven to be more potent than chloroquine, as no virus strain has been detected later in patients treated with hydroxychloroquine [82,88,89]. Thus, it could also be a potential drug to treat SARS-CoV-2 patients. Lopinavir and ritonavir are protease inhibitors that were used previously to treat HIV [90]. These drugs have been used against SARS-CoV and MERS-CoV, resulting in improved outcomes in patients [91,92]. Ritonavir and lopinavir combinations are also utilized against SARS-CoV-2. Improvements have been seen in Korean patients regarding the decrease in viral loads after intakes of ritonavir and lopinavir [93]. In Shanghai, improvements in pneumonia associated symptoms had also been seen in patients after ritonavir and lopinavir treatment [94]. The mortality rate and viral RNA detection were similar among patients treated with ritonavir and lopinavir as compared to for patients with no such treatment. Instead, ritonavir and lopinavir also show adverse effects in patients, as gastroenteritis was more prevalent in patients treated with these drugs. That is why this treatment was stopped after early trials due to its adverse effects on patients’ health [82]. However, in the same country, patients also recovered after ritonavir and lopinavir administration and a decrease in viral load was observed to the extent that no detectable viral concentrations were observed after treatment. It also helped patients to regain their normal body temperature [82]. Another study suggested that ritonavir and lopinavir treatment did not decrease the viral shedding process [82]. The mechanism behind this process of resistance is not understood yet. Some patients have shown improvement and others have developed different diseases after treatment with ritonavir and lopinavir. There may be mutations in the viral genome which make them resist antiviral treatments or there may be some other reasons for this outcome like patients’ clinical conditions and age factors. Further research is needed to understand the complete process. It has been reported that along with ritonavir and lopinavir, many other compounds might be useful for treating SARS-CoV-2 infection including nucleoside analogues, anti-inflammatory drugs (hormones and other molecules), neuraminidase inhibitors, RNA synthesis inhibitors (3TC and TDF), remdesivir, abidol, peptide (EK1), and Chinese traditional medicines such as LianHuaQingWen and ShuFengJieDu capsules [74].

### 9.2. Potential Therapeutic Compounds and Drugs

In addition to the drugs mentioned above, various other drugs will be developed against the coronavirus. Clinical trials are in progress to develop specific antiviral drugs and vaccines against SARS-CoV-2. Scientists are working hard on the development of a specific vaccine to control this pandemic. Some viral targeted agents may have the potential to work against SARS-CoV-2 in the future, as mentioned in Table 5. In addition to the abovementioned potential therapeutic agents and drugs, targeting cells inducing inflammatory response can also be an option for the treatment of SARS-CoV-2 patients. Inflammatory response leads to bursting of cytokines upon the onset of infection, consequently activating the innate immune response. One example is of toll-like receptors (TLRs) inducing signaling pathways upon activation, in turn leading to cytokines production and innate immune response. Targeting Toll-like receptors with some kind of antagonist (e.g., anti-TLR4-specific antibodies and TAK-242) would help to control the drawbacks associated with the genetic heterogeneity of SARS-CoV-2 [95,96]. Another option is to use immunosuppressants e.g., corticosteroids to treat the higher stage of infections. However, the use of immunosuppressants has its side effects. For instance, in influenza, immunosuppressants led to a higher death rate, superinfections, as well as long term viral exposure in patients [97]. In the case of MERS-CoV and SARS-CoV, the number of deaths was not increased but the virus was retained for a longer time in patients [98]. A better option is to target proteins that interact with the S (spike) proteins of the virus and allow them to enter the host cell. For example, type II transmembrane serine protease (TMPRSS2) is a host cell protease that activates spike proteins of the SARS virus. Targeting these proteases will lead to the development of the best therapy against SARS-CoV-2. For example, camostat mesylate is a serine protease inhibitor that suppresses and inhibits the enzymatic activity of TMPRSS2 [98]. However, in vivo studies are needed for a complete understanding of the TMPRSS2 antiviral mechanism. These are some of the potential therapies and treatment options that may prove beneficial to treat coronavirus. Diligent efforts are required for identifying an effective treatment against the virus. The vaccine could protect the public from getting infected, but once a person is affected, it could no longer help with treatment. Hence, there is a need to find out therapeutic targets in coronavirus. These targets can be proteins, enzymes, pathways, and receptors which are involved in the host-virus interaction.

### 9.3. Nutritional Treatment

Nutritional treatment is also an option for the treatment of patients infected with any virus. Vaccine/drug discovery and development is a time-consuming process and patients do not have enough time to wait. In the current scenario, nutritional treatment can be given to patients for quick recovery. Vitamin C/ascorbic acid is a powerful compound and is used to treat many viral diseases. Intake of vitamin C in prescribed quantities can help in the cure of disease and help patients to recover quickly. The vitamin C treatment has been successfully used against swine flu, bird flu, Chikungunya, and SARS in the past. It boosts the immune system and directly denatures/kills many human viruses. Vitamin C helps the immune system in fighting viruses, but its dosage is important. Even low doses help patients with a strong immune system. Intake of 200 mg vitamin C per day is reported as favorable for prevention, lessening disease complications and shortening the disease course, and allowing effective management of COVID-19 [104,105]. It also helps in treating the symptoms of severe respiratory illness [98]. Physicians of the Orthomolecular Medicine News Service review board recommended 3000 mg/day of vitamin C in various doses. It can be taken in the form of vinegar (Ascorbic acid) or sodium ascorbate, which is non-acidic. Appropriate agents as antiviral agents can be sprayed with nebulization quickly, thereby eliminating the virus in the pharynx area and wiping up the virus in the rest of the body. A favorable dose of hydrogen peroxide as 3% several times a day is also given as antiviral treatment [106]. Other recommended nutrients are zinc, magnesium, selenium, vitamin A, vitamin B complex, and vitamin D3. Magnesium is a key component of many biochemical pathways, and an intake of 400 mg/day should be included in the daily diet. Due to the lack of magnesium in food nowadays, it is supplemented in the form of chelate, citrate, chloride, or malate form. Viral infections like that of polio can be cured through magnesium more effectively than through vitamin C supplements [107]. Zinc is another nutrient that is essential in many biochemical pathways. It acts as an antioxidant and helps the body to fight against Swine flu, bird flu, and SARS [107]. A total of 20–40 mg/day dose of zinc is recommended for adults [108]. In the presence of selenium, the virus fails to mutate and immunocompetence is improved [109,110]. Nutritional treatment is not just an idea to cure diseases; realistically nutrients are necessary for the body to fight against viruses, as they enhance the overall immunity of the body [111].

## 10. Platforms for SARS-CoV-2 Vaccine Development

There are various platforms designed to develop vaccines against SARS-CoV-2, for instance, whole virion vaccines (inactivated and live attenuated), nucleic acid vaccines, protein subunit vaccines, and recombinant viral vaccines. 

Live attenuated vaccines (LAV) are viruses that can replicate through several passages from cell culture. On the other hand, inactivated vaccines are viruses that are inactivated by exposure to chemicals (e.g., formaldehyde) and heat [112]. Live attenuated vaccines are immunogenic and produce long-lasting immune responses in the host which protect against live pathogens [112,113]. Inactivated vaccines produce a weaker immune response and require more than one dose for effectiveness or some other adjuvants [114]. There are several safety issues regarding the use of attenuated vaccines, including the reactivation in vaccinated individuals, hence their use is not beneficial in the case of highly pathogenic viruses [112]. Attenuated vaccines are not suitable for individuals with a weak immune system, as they are more prone to infection if the virus reverts itself [115]. Moreover, preservation of LAVs requires ultra-cold temperatures and a sustained cold chain distribution system, which may not be available in all countries [114,115,116]. LAVs have undergone preclinical trials [117] and there is a company working on the development of a computationally designed virus that is non-pathogenic [118]. Another company is working on the formalin-inactivated SARS-CoV-2 virus and determined its efficacy in rhesus macaques [119]. This vaccine did not invite any pathological response; on the contrary, in the past, the LAV vaccine against SARS-CoV produced an eosinophil-derived immune response in mice. Histopathological changes were also observed in lungs derived from T helper 2 cells [120,121]. 

Protein subunit vaccines include antigenic proteins that are produced in vitro. Upon administration with adjuvants, these vaccines produce a strong humoral immune response and high immune memory [122]. Protein subunit vaccines include antigenic proteins and virus-like particles (VLPs), which produce many copies of antigens and produce strong immune responses without the help of adjuvants [122]. Worldwide institutions are working on protein subunit vaccines, since they are an attractive vaccine technology that does not require specific conditions for preservation [114]. However, the costs for mass production, specific mammalian optimization, and cell expression are important considerations [123,124]. 

Nucleic acid vaccines are produced by choosing specific proteins of pathogens (coding for their epitopes) to initiate an immune response in the host. These proteins are delivered into the host as DNA/RNA plasmid sequences [125,126,127]. Upon injection into host cells, the host cell machinery starts producing pathogen proteins which are recognized by the host immune system, in turn, initiating the production of antibodies against these proteins [127]. If RNA/DNA vaccine is non-capsulated, it will be rejected and removed from host cells soon after the injection. Advanced delivery technologies, such as encapsulation of RNA in liposomes, can be used to avoid the degradation of vaccines by host cells [128]. RNA vaccines produce T cell and antigen-specific antibody response against cancer in clinical trials. A mRNA-based vaccine known as the non-replicating rabies virus glycoprotein (RABV-G) stimulates stable and potent neutralizing antibodies in domestic pigs and mice. It produces functional antibodies against glycoprotein of rabies virus [115,129]. DNA vaccines are also immunogenic in the case of animals; however, in humans, they show a weak immune response and nesed multiple doses with adjuvants [130]. Four DNA vaccines are available for animal use but currently, there are no licensed DNA and RNA vaccines for humans [131]. Nucleic acid vaccines have been developed and some are still under development to protect against SARS-CoV-2 by several institutes such as Moderna (Cambridge, MA, USA), Inovio Pharmaceuticals (Plymouth Meeting, PA, USA), Pfizer (New York, NY, USA), the Imperial College London, Takis Biotech (Rome, Italy), and BIOCAD (St. Petersburg, Russia). The vaccine named BNT162b2 has been approved for delivery and sale in multiple countries. The studies carried out on this RNA vaccine to check its safety measures and immunogenicity in adults (from 18 to 55 years old), who were given 2 doses of vaccine with a 21 days interval, have revealed that neutralizing titers of SARS-CoV-2 and IgG concentrations binding with RBD (receptor binding domain) increased 1.9–4.6 fold in COVID-19 convalescent sera obtained at least 14 days after positive PCR [132]. Results of these studies support the public use of the mRNA vaccine [133]. Nucleic acid vaccines are easy to produce and relatively cheap with the possibility of mass production [134]. The minimum time required for a vaccine development and for its clinical trials is almost 1 year. 

Last but not least, another platform for vaccine development is Recombinant viral vector vaccines. These vaccines contain live viruses that can replicate themselves, but they are engineered to carry some extra pathogenic genes of interest. These genes produce proteins after the injection of vaccines into the host. The host immune system produces antibodies against these proteins [135]. Challenges in the development of these vaccines elicit an immune response against the vector instead of an antigen-specific response, as well as the loss of extra viral genes during recombination procedures and during replication inside host cells. However, pre-clinical and clinical trials showed that one dose of such types of vaccines is enough to elicit an effective immune response [136]. Human adenoviruses (hAds) are potential recombinant viral vectors. However, due to their frequent spread in the population, pre-existing immunity makes vaccines less efficient [137,138]. Chimpanzee adenovirus (ChAd) was developed, which is more efficient than hAds due to its low prevalence, hence, acting as a neutralizing antibody [139,140]. Preclinical studies show that ChAd vectors are 100% efficient against emerging viruses and a single dose is sufficient to produce rapid immunity in individuals [138,141,142]. 

## 11. Current Status of COVID-19 Vaccine Development 

Vaccination produces everlasting immunity in the host by introducing it to antigens and produce a kind of immunologic memory before encountering pathogens. SARS-CoV-2 gains entry into the host by its surface spike (S) protein [143]. This protein attaches with ACE2 (angiotensin-converting enzyme 2) receptor on host cells. These receptors are found abundantly on pulmonary epithelial cells [115]. Currently, vaccines are designed to target S protein on the viral surface. Just after the viral entry in the host, the S protein is recognized by the host immune system, which produces antibodies against both the S protein and nucleoprotein. The first genomic sequence of SARS-CoV-2 was published in January 2020, triggering global activity to develop a vaccine against the contagious virus. The first SARS-CoV-2 vaccine candidate was approved for human clinical testing on 16 March 2020. The Coalition for Epidemic Preparedness Innovations (CEPI) is collaborating with vaccine developers and global health authorities to develop a vaccine against SARS-CoV-2 [144]. On 8 April 2020, the global SARS-CoV-2 vaccine R&D landscape consisted of 115 vaccine candidates. Out of these 115 candidates, 37 were unconfirmed and 78 were confirmed and active. Platforms for vaccine development depend on mRNA and DNA providing potential and flexibility for antigen manipulation. The vaccines based on viral vectors often provide high protein expression, induce a strong immune response, and are highly stable. Vaccine developers (GlaxoSmithKline, Dynavax, and Seqirus) planned to use adjuvants as vaccines because of their ability to enhance immunity and the viability of low doses [144]. According to WHO, six vaccines were approved/authorized against SARS-CoV-2 for public use before 15 December 2020. These vaccines have been designed by using different techniques such as a m-RNA based vaccine (BNT162b2), Peptide vaccine (EpiVacCorona), inactivated virus vaccine (BBIBP-CorV and CoronaVac), and non-replicating viral vector (Sputnik V) vaccine. The SARS-CoV-2 has numerous variants, and it can mutate in an individual and a population over time. Therefore, many questions about the efficacy of vaccines rise with time. It has been reported that the maximum variants have a mutation in the D614G region and the ACE2 binding site is not affected. So, it can be stated that the vaccines developed for SARS-CoV-2 would be effective against its variants [145]. Some of the authorized/approved and candidate vaccines have been discussed in Table 6.

## 12. Recommended Preventive Measures

Since SARS-CoV-2 is of a zoonotic origin, extensive measures are required to control person to person transfer and the rapid spread of the disease. Despite strict regulations, SARS-CoV-2 has spread very quickly globally. Special attention, care, and efforts are needed to reduce transmission among susceptible populations, including elder people, children, and health care workers. Preventive measures are the current master plan to reduce the number of active cases. A guideline was published for health care providers, researchers, medical staff, and the general public [146]. The early spread of disease was among the elderly population due to their weak immune systems and rapid onset of illness [147,148]. Various public services have provided decontaminating agents to people for hand cleansing on a routine basis. Care should be taken among health care societies while care should be exercised in dealing with virus samples such as urine and fecal samples that provide an alternative route of transmission [149,150]. Major preventive control measures have been implemented like the screening of travelers to minimize disease spreading all over the world. 

Some general recommendations are issued by WHO as well as other organizations:

1. Regular use of face masks [151].
2. Wash your hands frequently and sanitize them after close contact with objects and patients. Isolate patients in a separate room and minimize visits to patients [152].
3. Avoid personal contact with farm and wild animals [152].
4. Avoid close contact with people that have any respiratory illness or symptoms [153].
5. Specifically, people with weak immune systems should avoid public gatherings and healthy people should also avoid gatherings to minimize the chances of getting the disease [154].
6. People with flu and a cough should avoid close contact with healthy people. While coughing and sneezing they should use disposable tissue/cloth and dispose of them properly. Afterward, wash hands frequently and use sanitizer [155].
7. Strict hygiene rules should be followed in hospitals and other health care departments to avoid the spread of disease and to prevent infection [156].
8. Some of the vaccines like Pfizer-BioNTech COVID-19 Vaccine and Moderna COVID-19 Vaccine are approved for emergency use; however, they have not been fully evaluated for efficacy against SARS-CoV-2 variants that recently emerged in the UK and South Africa [157].

The most important strategies which people should follow is to avoid public gatherings, use portable hand sanitizers, and avoid contact with the face, mouth, and nose after visiting contaminated areas such as hospitals and other health care units. For health care workers, care should be taken by wearing gloves, masks (FFP3 and N95), gowns, and eye protection to avoid transmission of the virus.

Since there is no proper treatment of SARS-CoV-2 available to everyone yet, only by following SOPs and by exercising personal care can the chances of getting an infection can be minimized. The general public should take vitamin-rich foods to enhance body immunity. 

## 13. Conclusions

Re-emergence of the virus after modifications in its genome for stable adaptation is a serious concern to human health. SARS-CoV-2 has a zoonotic origin and changed its host from bats and animals to humans. In 2019, it appeared for the very first time, transmitted from human to human, and gradually spread to over 200 countries. This virus spread through close human contact by coughing, sneezing, and aerosols. The transmission was so rapid and widespread that controlling it became a difficult task. Through sequencing and phylogenetic analysis, researchers found that this virus is novel and there is no cure for its infection. Its genome has similarities with those of SARS-CoV and MERS-CoV but due to mutations somewhere in the genome, it became a novel virus. It causes infections with mild symptoms (fever, flu, and cough) to severe symptoms and clinical outcomes (ARDS, septic shock, respiratory failure, and Multi-organ dysfunction). There are multiple risk factors involved regarding the severity of disease like comorbidities, organ failures, diabetes, etc. The ratio of illness and deaths has been high among elder people due to having a weaker immune system. There is no proper treatment and no specific drugs against this virus to date. Although some vaccines have been approved/authorized against the virus, preventive strategies should still be adopted by the public to minimize the chance of getting the disease.

## Figures and Tables

**Table 1:** Sequence homology between SARS-CoV-2 and other coronaviruses strains [7].

Coronaviruses Strains	Sequence Similarity
SARSr-CoV; RaTG13	96.20%
bat-SL-CoVZC45	88.00%
bat-SL-CoVZXC21	88.00%
SARS-CoV	82.45%
SARS-HCoV Tor2	82.00%
SARS-HCoV BJ01	82.00%
MERS-CoV	69.58%
HCoV-OC43	68.93%
HCoV-HKU1	67.59%
HCoV-229E	65.04%
HCoV-NL63	65.11%

**Table 2:** Percentage identity between proteins of SARS-CoV-2 and the Coronaviridae family [31].

Gene	SARS NC_004718.3	Bat MG772934.1	Bat DQ022305.2
ORF1ab	86.12%	95.15%	85.78%
ORF3a	72.36%	92.00%	72.99%
ORF6	68.85%	93.44%	67.21%
ORF7a	85.25%	88.43%	88.52%
ORF7b	81.40%	93.02%	79.07%
ORF8	30.16%	94.21%	57.02%
ORF10	72.45%	73.20%	74.23%
S (Spike)	75.96%	80.32%	76.04%
E (Envelope)	94.74%	100%	94.74%
M (Membrane)	90.54%	98.65%	90.99%
N (Nucleo-capsid)	90.52%	94.27%	89.55%

**Table 3:** Percentage of risk factors between survivors and non-survivors (associated with the severity of SARS-CoV-2) [47].

Risk Factors	Total Patients (191)	Survivors (137)	Non-Survivors (54)
Comorbidity	91 (48%)	55 (40%)	36 (67%)
Hypertension	58 (30%)	32 (23%)	26 (48%)
Diabetes	36 (19%)	19 (14%)	17 (31%)
Coronary heart disease	15 (8%)	2 (1%)	13 (24%)
Chronic obstructive lung disease	6 (3%)	2 (1%)	4 (7%)
Carcinoma	2 (1%)	2 (1%)	0
Chronic kidney disease	2 (1%)	0	2 (4%)
Other	22 (12%)	11 (8%)	11 (20%)

**Table 4:** Differentiation of various biomarkers among survivors and non-survivors.

Laboratory Markers	Survivors	Non-Survivors
Lymphocytes count	Initially low but with hospitalization (after 7 days) it improved	Lymphopenia observed (a low number of lymphocytes)
Blood d-dimer levels	Normal level	A very high level which increased with worsening of the disease
High-sensitivity cardiac troponin I	Normal level	A very high level (after 16 days of disease onset) which increased with worsening of the disease.
Serum ferritin	Normal level	A very high level which increased with worsening of the disease
Lactate dehydrogenase	Increased with the early onset of illness but normalized/decreased after 13 days.	A very high level which increased with worsening of the disease
IL-6	Normal level	A very high level which increased with worsening of the disease
SOFA	Low	High

**Table 5:** Potential therapeutic compounds and drugs for SARS-CoV-2 treatment (in trials).

Antiviral Compounds	Drug’s Status	Compound’s Functions to Inhibit Viral Action	References
Favipiravir (T-705), a guanine analogue	Approved for influenza treatment	Effectively inhibits the RNA-dependent RNA polymerase of RNA viruses such as influenza, Ebola, yellow fever, chikungunya, norovirus, and enterovirus.	[98]
Favipiravir+baloxavir marboxil, favipiravir+ interferon-α	An approved influenza inhibitor	Targeting the cap-dependent endonuclease.	[99]
Ribavirin (guanine derivative)	Approved for treating HCV and respiratory syncytial virus (RSV) that has been evaluated in patients with SARS and MERS	The mechanism is not understood yet.	[100]
Remdesivir (GS-5734), phosphoramidate prodrug of an adenine derivative	Approved HIV reverse transcriptase inhibitor	Has broad-spectrum activities against RNA viruses such as MERS and SARS in cell cultures and animal models, and has been tested in a clinical trial for Ebola; it also inhibits SARS-CoV-2 in vitro [99], and patients recovered in the US after administration [101].	[99]
Galidesivir (adenosine analogue)	Approved (originally developed for HCV, also has shown antiviral activities in preclinical studies against many RNA viruses, including SARS and MERS).	The mechanism is not understood yet.	[100]
Disulfiram (Protease inhibitor)	An approved drug to treat alcohol dependence.	Inhibit the papain-like protease of MERS and SARS.	
Lopinavir and ritonavir	Approved HIV protease inhibitors.	Inhibit the 3-chymotrypsin-like protease of SARS and MERS.	[101]
Griffithsin (red algae-derived lectin)	Approved to treat HIV.	Binds to oligosaccharides on the surface of various viral glycoproteins, including HIV glycoprotein 120 and SARS-CoV spike glycoprotein.	[100]
Pegylated interferon alfa-2a and -2b	Approved to treat HCV and HBV.	Stimulate innate antiviral responses in patients infected with 2019-nCoV.	[99]
Chloroquine	Approved immune modulator.	Triggers the Glycosylate viral cell’s receptors and increases endosomal PH while also acting as autophagy inhibitors.	[84]
Nitazoxanide	Approved for diarrhea treatment.	The mechanism is not understood yet.	[94]
Monoclonal (immunoglobulin G1 (MHAA4549A, VIS410) and polyclonal antibodies (SAB-301)	Approved for influenza, however, trials are continuing against SARS-CoV-2.	Several antibodies have been shown to bind influenza virus haemagglutinin and inhibit virus replication.	[102]
Convalescent sera (prepared from a patient’s blood, acts as a type of passive immunization)	Approved (Target cytomegalovirus, hepatitis B virus, and varicella-zoster virus).	The mechanism has not been described yet.	[94]
Nafamostat	Potent against MERS-CoV	Prevents membrane fusion	
Pathways inhibitors (Fedratinib, Sunitinib, Baricitinib, and Erlotinib)	Approved for medical use.	Inhibits the AAK (AP2-associated protein kinase 1) pathway, which involves endocytosis. Baricitinib also inhibits cyclin G-associated kinase, which is another regulator of endocytosis.	[103]

**Table 6:** Some of authorized/approved and candidate vaccines against SARS-CoV-2 (more details can be found at: https://www.who.int/emergencies/diseases/novel-coronavirus-2019/covid-19-vaccines (accessed on 1 February 2021).

Type of Platform	Name of Candidate Vaccine	Doses	Manufacturer	Status	Countries Authorized for Emergency Use/Trials
Non-replicating viral vector	Ad5-nCoV	1	CanSino Biological Inc./Beijing Institute of Biotechnology	Approved	China and Mexico
	Sputnik V	2	Gamaleya Research Institute; Health Ministry of the Russian Federation	Approved	Russia, Belarus, Argentina, Hungary, UAE, Algeria, Bolivia, Serbia, Palestinian territories, and Iran
	AZD1222	2	AstraZeneca + University of Oxford	Approved	UK, Argentina, El Salvador, India, Mexico, Bangladesh, the Dominican Republic, Pakistan, the Philippines, Nepal, Brazil, and Sri Lanka
	Ad26.COV2-S	2	Janssen Pharmaceutical	Phase III	USA, Brazil, Chile, Colombia, Mexico, Peru, South Africa, Ukraine, and the Philippines
Inactivated virus	BBIBP-CorV	2	Sinopharm + China National Biotec Group Co + Wuhan Institute of Biological Products	Approved	Bahrain, China, Egypt, Iraq, Jordan, Pakistan, Seychelles, and the UAE
	CoronaVac	2	Sinovac Research and Development Co., Ltd.	Approved	China, Indonesia, Brazil, and Turkey
	WIBP	2	Sinopharm + Wuhan Institute of Biological	Phase III	China
	Covaxin	2	Bharat Biotech International Limited	Approved	India
RNA	Comirnaty (BNT162b2)	2	Pfizer/BioNTech + Fosun Pharma	Approved	The UK, Europe, Argentina, Australia, Bahrain, Canada, Chile, Costa Rica, Ecuador, Hong Kong, Iraq, Israel, Jordan, Kuwait, Mexico, Oman, Panama, the Philippines, Qatar, Saudi Arabia, Singapore, the UAE, and the USA
	mRNA-1273	2	Moderna + National Institute of Allergy and Infectious Diseases (NIAID)	Approved	Austria, Belgium, Bulgaria, Canada, Croatia, Cyprus, Czech Republic, Denmark, Estonia, Finland, France, Germany, Greece, Hungary, Iceland, Ireland, Israel, Italy, Latvia, Liechtenstein, Lithuania, Luxembourg, Malta, Mongolia, Netherlands, Norway, Poland, Portugal, Romania, Seychelles, Slovakia, Slovenia, Spain, Sweden, Switzerland, the UK, and the USA
Protein subunit	NVX-CoV2373	2	Novavax	Phase III	The UK, the USA
	ZF2001	3	Anhui Zhifei Longcom Biopharmaceutical + Institute of Microbiology, Chinese Academy of Sciences	Phase III	China
	EpiVac-Corona	2	Federal Budgetary Research Institution State Research Center of Virology and Biotechnology Russia	Approved	Russia

## Data Availability

The data presented in this study are available within the article.
